# Annotations

**Published:** 1901-06-29

**Authors:** 


### Annotations.

### Smoke Nuisances.

According to the Public Health (London) Act, 1891, "any chimney (not being the chimney of a private dwelling-house) sending forth black smoke in such a quantity as to be a nuisance," is a nuisance, and may be abated summarily. In a recent prosecution, however, an attempt was made to throw upon the prosecution the onus of proving not only that black smoke was emitted, and that this smoke was sufficiently dense to be a nuisance, but that it had as a fact injured or annoyed some individual. Ten summonses had been issued against the South London Electric Supply Corporation for allowing black smoke to issue from a chimney on their works during last November, but although there was evidence as to the issue of the smoke and as to its blackness, no one was called to prove that he had been injured or annoyed by this smoke, the prosecution having apparently relied upon the generally accepted interpretation that black smoke so issuing from a manufacturing chimney is *de facto* a nuisance. The magistrate convicted and imposed a fine of £10 for each day, but a case was stated to determine whether evidence of the mere issuing of such black smoke was sufficient, or whether it was necessary also to produce evidence that it had been a nuisance to someone. It was argued for the appellant that the words in the Act, namely, "in such a quantity as to be a nuisance" made it necessary to call evidence that it had, in fact, been a nuisance to someone. The case came before Lord Alverstone and Mr. Justice Lawrance on May 6th, when, according to the report in the *Law Journal*, the court held that the mere proof of the issue of smoke did not compel the magistrate to convict, but that it was not necessary to show injury or nuisance to any particular individual. Thus we come back to the old and generally received view, with, however, this important proviso that the magistrate must exercise his discretion.

### Christian Scientists and Life Assurance.

The Christian Scientists in England are a sect to laugh at rather than to take seriously. It is different in America where they are sufficiently numerous to be at least a nuisance. Amidst the maze of indefinite assumptions and incomprehensible dogmas on which their faith is built there is at least one point which is fairly positive, and that is their rejection of the assistance of medical science in the treatment of disease. So far as the individuals are concerned this might well be a matter of indifference to the rest of the world. The law, of course, must be called in to protect children and help other helpless persons, but there seems no reason why adults should be prevented from suffering the penalties exacted by their religion—unless, indeed, we adjudge them all insane—in which case the law again would intervene for their protection. But when we leave the individual and remember that a large number of Christian Scientists, although cutting themselves adrift from the advantages given by modern knowledge, continue to claim the benefits of modern co-operation, new problems arise. Just now the question which has arisen is in regard to life assurance. The stability of any life assurance company depends, among other things, upon its customers continuing to be drawn from the same class of people, that is, from among people of at least as long an average duration of life as those from whose lives its tables were calculated. But in mutual offices something else besides stability has to be considered. One of the advantages of the mutual offices is that if the assured live on an average longer than it was calculated that they would do, and if therefore the business becomes very successful, a certain portion if not all of this extra profit comes back to them in the form of bonus, and no one doubts that the amount of bonus distributed is one of the great attractions which draws people to certain offices. These bonuses depend to a large extent upon a careful selection of lives. Everyone sees for example that a mutual office which has the greater proportion of its clients engaged in long-lived professions, such as the church, can afford to accept a smaller annual premium than could be accepted by an office which accepted a large number say of publicans at its ordinary rates; and the subscribers to a long-lived mutual office have good cause for complaint if its managers take to accepting a lot of shaky lives at the same rate as they are paying. Here comes in the Christian Scientist, for unless we are to admit that physicians are charlatans and that medicine has no efficacy in the treatment of disease, it is obvious that any sect which deliberately rejects such a means of prolonging the life of its members as is offered by medical treatment in case of illness, must, so far as life assurance is concerned consist of risky lives. Hence the trouble. People in America, when choosing an office, are beginning to ask which offices accept Christian Scientists at ordinary rates, feeling sure that any large introduction of such an element of weakness among the assured must tell upon the profits as years go on. This certainly is a view which requires careful consideration.

### Unnecessary Post-Mortem Examinations.

We have often commented upon the vagaries of coroners and their juries, and especially upon their neglect of post-mortem examinations in cases in which without them it is absolutely impossible to ascertain the cause of death. Again and again it happens that verdicts of death from natural causes, convulsions, or what not, are returned in cases on which no post-mortem examination has been made, and in regard to which, to say the least of it, no evidence has been offered either to show the absence of poison or injury, or to prove what was the actual cause of death. While, however, we point out the errors of others, let us not be oblivious of our own, for it may fairly be suggested that medical men are sometimes rather apt to strain the importance and necessity of the post-mortem, and to pretend, for surely it is a pretence, that without an internal examination they can tell nothing about the cause of death. A somewhat extreme instance of this sort of thing happened at a recent inquest respecting the death of a stoker who was killed by an explosion on board the torpedo-destroyer *Daring*. Evidence was given to the effect that there had been a sudden explosion in the stokehold, that four men who were injured escaped, but that the deceased was found lying at the bottom of the ladder, that the stokehold was full of steam, that there was great difficulty in getting at the body as there were four inches of scalding water on the stokehold plates, and the doctor gave evidence that when he arrived the man was dead, having been scalded over the greater part of the body. Notwithstanding all this, however, according to the report, the doctor "could not certify to the cause of death until he had made a post-mortem examination."

